# The Development of Gelatin/Hyaluronate Copolymer Mixed with Calcium Sulfate, Hydroxyapatite, and Stromal-Cell-Derived Factor-1 for Bone Regeneration Enhancement

**DOI:** 10.3390/polym11091454

**Published:** 2019-09-05

**Authors:** Yun-Liang Chang, Chia-Ying Hsieh, Chao-Yuan Yeh, Feng-Huei Lin

**Affiliations:** 1 Department of Biomedical Engineering, National Taiwan University, No. 1, Sec.1, Jen-Ai Road, Taipei City 10051, Taiwan; 2 Department of Orthopaedic Surgery, National Taiwan University Hospital, No. 7, Chung Shan South Road, Taipei City 10002, Taiwan; 3 Integrative Stem Cell Center, China Medical University, No. 2, Yude Road, Taichung City 40447, Taiwan

**Keywords:** SDF-1, bone defect, bone regeneration, mesenchymal stem cells, gelatin, hyaluronate, calcium sulfate, hydroxyapatite, biomaterial

## Abstract

In clinical practice, bone defects still remain a challenge. In recent years, apart from the osteoconductivity that most bone void fillers already provide, osteoinductivity has also been emphasized to promote bone healing. Stromal-cell-derived factor-1 (SDF-1) has been shown to have the ability to recruit mesenchymal stem cells (MSCs), which play an important role in the bone regeneration process. In this study, we developed a gelatin–hyaluronate (Gel-HA) copolymer mixed with calcium sulfate (CS), hydroxyapatite (HAP), and SDF-1 in order to enhance bone regeneration in a bone defect model. The composites were tested in vitro for biocompatibility and their ability to recruit MSCs after material characterization. For the in vivo test, a rat femoral condyle bone defect model was used. Micro computed tomography (Micro-CT), two-photon excitation microscopy, and histology analysis were performed to assess bone regeneration. As expected, enhanced bone regeneration was well observed in the group filled with Gel-HA/CS/HAP/SDF-1 composites compared with the control group in our animal model. Furthermore, detailed blood analysis of rats showed no obvious systemic toxicity or side effects after material implantation. In conclusion, the Gel-HA/CS/HAP/SDF-1 composite may be a safe and applicable material to enhance bone regeneration in bone defects.

## 1. Introduction

Bone defect is a common clinical scenario during treatment of bone-related pathology. Comminuted fractures, neoplasms, and bone infections are common causes of bone defect [1]. Apart from autografts or allografts, different kinds of synthetic bone substitutes have been used by clinical doctors for decades to treat bone defects [2,3,4]. However, each kind of material still has its own limitations.

Calcium sulfate (CS) has been used to treat bone defects since 1892 and is still presently useful for different pathologies [5,6]. CS is cheap, can be synthesized easily, and has osteoconductivity and good mechanical strength [7]. However, the rapid resorption of CS may cause some problems, including serious wound discharge or delayed bone union [8,9]. Hydroxyapatite (HAP) and tricalcium phosphate (TCP) are both osteoconductive and have been widely used for bone defects [10,11]. Hyaluronate (HA) has a relatively poor resorption rate compared with CS, and TCP falls in between. It takes 6–18 months for TCP and up to 10 years for HAP to be completely resorbed in human beings. However, due to the lack of osteoinductivity, all these ceramics are not suitable for use in areas with poor vascular perfusion or large segmental defects.

Bone morphogenetic proteins (BMPs) such as BMP-2 or BMP-7 are also used to treat bone defects, as their osteoinductivity can promote bone repair [2,3,4,12]. However, BMPs are very expensive and have some pro-oncogenic concerns. Demineralized bone matrix (DBM) has become increasingly popular due to its osteoinductivity and the various forms it can take for different clinical needs [13,14]. DBM is made from allograft with the growth factors remaining, and it is suitable for filling bone defects due to its collagen scaffold [15,16]. It is also slightly cheaper than BMPs and has no pro-oncogenic concerns. However, the potential for disease transmission is still a problem due to its allograft-derived nature.

In the management of bone defects, the osteoconductivity and osteoinductivity of bone void fillers are equally important. Using a synthetic bone substitute seems to be a better way to avoid potential disease transmission, but most bone substitutes lack osteoinductivity. Stromal-cell-derived factor-1 (SDF-1) is a dominant chemokine in bone marrow which can be induced in the periosteum of injured bone [17]. In addition to many signaling functions in different organs, it is able to recruit mesenchymal stem cells (MSCs) [18,19]. Due to the potential benefit of MSCs in bone regeneration, SDF-1 may be considered to have a certain degree of osteoinductive effect, resulting in enhanced bone growth and new bone formation [20,21]. Gelatin (Gel) is a denatured collagen and hyaluronate (HA) is a non-sulfated glycosaminoglycan. Both of them have good biodegradability and biocompatibility. Gel can enhance cell proliferation, differentiation and attachment, while HA may influence cell mobility, cell–matrix adhesion and cell–cell interaction [22,23]. By mixing Gel and HA together, it can create a biocompatible and biodegradable scaffold to contain various bioactive agents or materials. In this study, we integrated a gelatin–hyaluronate (Gel-HA) copolymer with CS and HAP particles after being crosslinked by 1,4-butanediol diglycidyl ether (BDDE), as these provide good mechanical properties as bone fillers and drug carriers. SDF-1 was also added in order to enhance MSC recruitment and to test the possible bone growing effect. We hypothesized that this Gel-HA/CS/HAP composite with SDF-1 may promote bone healing in a bone defect animal model.

## 2. Materials and Methods

### 2.1. Materials

Several materials were purchased from Sigma-Aldrich (St. Louis, MO, USA), including gelatin type A from porcine skin (Gel), hyaluronic acid sodium salt (HA), BDDE, calcium sulfate hemihydrate (CS), and calcium hydroxide. SDF-1 was purchased from PeproTech (Rocky Hill, NJ, USA). Human umbilical cord blood mesenchymal stem cells immortalized by human telomerase reverse transcriptase (cbMSC-hTERT)s were obtained from the American Type Culture Collection (ATCC).

### 2.2. Preparation of Hydroxyapatite

To obtain the optimal particle size, hydroxyapatite was made in our own laboratory. Phosphoric acid (0.3 M) was added dropwise to a 0.5 M calcium hydroxide solution at the rate of 3 mL/min, and the pH value was adjusted to 8.5. The mixture was then stirred for 2 h at 85 °C and left standing at 85 °C for 24 h. After that, the precipitated powder was collected. The powder was then washed three times with double-distilled water and freeze-dried [24,25].

### 2.3. Preparation of Gel-HA/CS/HAP Composite with SDF-1

First, Gel (10 wt %, 300 Bloom) and HA (0.5 wt %) were dissolved in distilled water above 37 °C, respectively. At the volume ratio of 85:15, the Gel solution and HA solution were well mixed. In the meantime, BDDE (0.5 vol %) was added to the mixture as a crosslinker. Using a magnetic stirrer, the mixture was mixed for 24 h at 37 °C. After that, CS and HAP were mixed at the ratio of 50:50 as an inorganic mixture. At the weight ratio of 75:25, the crosslinked organic mixture (Gel and HA) and inorganic mixture (CS and HAP) were mixed. Then, 100 ng/mL of SDF-1 was added to the mixture after the temperature of the mixture dropped to room temperature. The final mixture was then poured into a petri dish and kept at 4 °C.

### 2.4. Material Characterization

#### 2.4.1. X-ray Diffraction (XRD) Analysis

X-ray diffraction (Rigaku, TTRAX 3, Tokyo, Japan) was used to characterize the composition of the Gel-HA/CS/HAP composite. The tension of the XRD was set to 30 kV, and the current was 20 mA. The scanning rate was 10°/min, while the scanning degree was set to 2θ = 10°–80°.

#### 2.4.2. Fourier-Transform Infrared Spectroscopy Analysis

Using Fourier-transform infrared spectroscopy (Jasco, FT/IR-4200, Tokyo, Japan), the success of Gel and HA crosslinking with BDDE was determined. 

#### 2.4.3. Swelling Ratio

At 37 °C, the *swelling ratio* of the Gel-HA/CS/HAP composite was determined by incubation in phosphate buffer saline (PBS, pH 7.4). The composite with a known weight (*W*_d_) was placed in PBS for various time points. After the surface-adsorbed water was removed by filter paper, the wet weight (*W*_s_) of the composite was obtained. The *swelling ratio* was calculated as follows:

(1) $$Swelling\;ratio\;\left(\%\right)=\frac{W_{s}-W_{d}}{W_{d}}\times 100\%$$

#### 2.4.4. Degradation Test

For the in vitro degradation test, the Gel-HA/CS/HAP composite was immersed in PBS solution (pH 7.4) at 37 °C. The degraded gelatin solution was measured by an ELISA reader (Tecan, Sunrise, Melbourne, Australia) at 230 nm at various time points. 

#### 2.4.5. SDF-1 Release Profile

Considering the cost of SDF-1, we chose fluorescein isothiocyanate (FITC) conjugated insulin as the model drug. Since insulin (6 kDa) shares a similar molecular weight and shape with SDF-1 (8 kDa), it may also present a similar release profile to SDF-1. The Gel-HA/CS/HAP composite with FITC-insulin was immersed in PBS solution (pH 7.4) at 37 °C. Then, the released FITC–insulin solution was measured by an ELISA reader at 488 nm at various time points.

### 2.5. In Vitro Study

#### 2.5.1. Cell Culture

To culture the cbMSC-hTERT cells, **α**-minimum essential medium (MEM) supplemented with 20% fetal bovine serum (FBS), 1% penicillin/streptomycin/amphotericin B, 1.0 mM sodium pyruvate, 4 ng/mL recombinant human basic fibroblast growth factor (rHubFGF), and 30 μg/mL hygromycin was used. Under an atmosphere of 5% CO_2_, cbMSC-hTERT cells were culture in a humidified incubator at 37 °C [26].

#### 2.5.2. Cell Viability

To test cell viability, cbMSC-hTERT cells (10^4^ cells/well) were cultured on a 96-well plate for 24 h. Then, 100 μL of the material extracts (0.2 g material/mL media) were added into the wells and incubated for 24 h. After that, 100 μL of water-soluble tetrazolium salt (WST-1) solution was added to the wells and incubated for 2 h. Using an ELISA reader, the absorbance values of each well were measured at 450 nm.

#### 2.5.3. MSC Recruitment Test

cbMSC-hTERT cells (10^6^ cells/well) were cultured on a six-well plate for 24 h. Using cell scrapers, half of the cells were then removed. In the area without cells, the Gel-HA/CS/HAP composites with or without SDF-1 were prepared and placed into the six-well plate. The migration of cbMSC-hTERT cells was observed at 0, 24, 48, and 72 h.

### 2.6. In Vivo Study

#### 2.6.1. Implantation of Composite in Rat Femur Bone Defect Model

Eighteen Wistar male rats (300 g) were purchased commercially and kept in different cages in groups of three. Water and food were provided properly. To acclimate them to the environment, these animals were first kept a week in an animal house. Before operation, the rats were anesthetized with isoflurane. The fur around the left lower limb was gently shaved, and the skin was disinfected with alcohol. A midline skin incision was made over the left knee, and the medial femoral condyle was exposed. A bone defect that was 2.5 mm in diameter and 5 mm in depth was created over the medial femoral condyle by a trephine drill [27,28,29,30,31]. The Gel-HA/CS/HAP composite without SDF-1 and the composite with 100 ng/mL of SDF-1 were prepared and filled into the bone defect based on the previously assigned group. The bone defect was left in situ without any implant in the control group. The incision was then closed with 4–0 sutures. The animals were allowed to recover and were kept under proper care after operation. Three rats of each group were sacrificed one or two months after implantation. The whole femur bone specimen was collected gently. All animal studies were performed according to the protocol approved by the Institutional Animal Care and Use Committee (IACUC) of the National Taiwan University College of Medicine and College of Public Health.

#### 2.6.2. Blood Analysis

Before sacrifice, a cardiac puncture was performed with a 23G needle under anesthesia. The obtained blood sample was divided between two different tubes. One tube was centrifuged, and serum was extracted for biochemical analysis. The other tube contained dipotassium ethylenediaminetetraacetic acid (K2EDTA), which was sent for whole blood analysis. For the biochemical test, lactate dehydrogenase (LDH), alkaline phosphatase (ALKP), and Ca were measured. For the whole blood test, red blood cells (RBCs), hemoglobulin (HGB), hematocrit (HCT), mean corpuscular volume (MCV), mean corpuscular hemoglobin (MCH), mean corpuscular hemoglobin concentration (MCHC), white blood cells (WBCs), neutrophil (NEUT), lymphocyte (LYMPH), monocyte (MONO), eosinophil (EO), and basophil (BASO) were measured.

#### 2.6.3. Two-Photon Excitation Microscopy

Right after sacrifice, fresh femur specimens were inspected under two-photon excitation microscopy. Using an 890 nm laser beam as the excitation light source, the microscope received a double frequency of 445 ± 10 nm to generate a signal to observe type I collagen of bone tissue and the surrounding soft tissue. At the same time, it received a spontaneous fluorescent signal of 500–530 nm from the specimens.

#### 2.6.4. Micro-CT

For the micro-CT analysis, the obtained femur bone specimens were fixed in 10% formaldehyde for a week and then transferred to 95% ethanol. The distal part of the femur was scanned by a Bruker SkyScan 1076 micro-CT. The reconstructed micro-CT data were analyzed to determine the amount of bone and the presence of the implants and to calculate the bone volume/tissue volume (BV/TV). DataViewer and CTAn softwares (Bruker, Billerica, Massachusetts, USA) were used to make 2D and 3D images, respectively.

#### 2.6.5. Histological Analysis

Femur specimens were first fixed with 10% formaldehyde for a week and then decalcified with 5% nitric acid for three days. After decalcification, the specimens were dehydrated through a sequential alcohol (70–100%) treatment. The dehydrated specimens were then transitioned to xylene series and eventually paraffin for embedding. Paraffin-embedded samples were then cut into 5-µm sections and attached to glass slides. The sections were stained with hematoxylin and eosin (H&E) and Masson’s trichrome (MT) to visualize the dense tissue and new bone formation.

## 3. Results and Discussion

### 3.1. XRD Analysis

**Figure 1:**
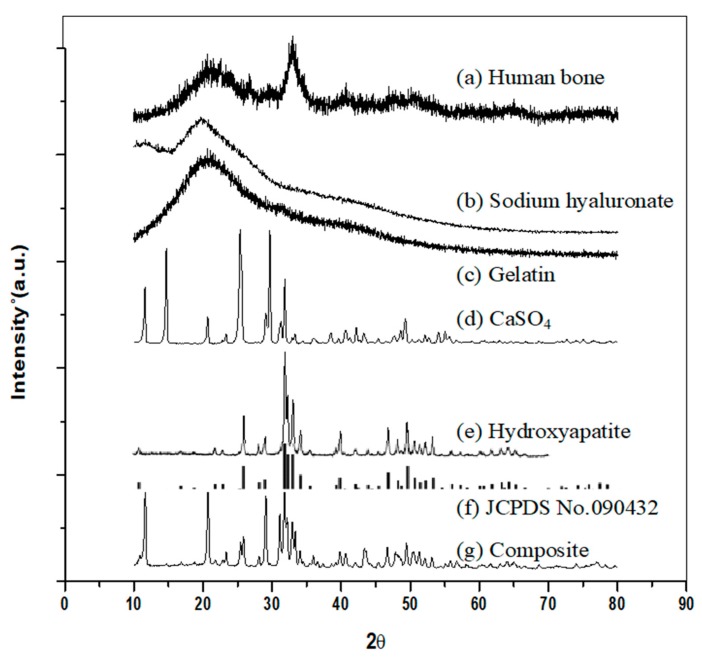
X-ray diffraction (XRD) patterns: (**a**) human bone, (**b**) sodium hyaluronate, (**c**) gelatin, (**d**) calcium sulfate, (**e**) hydroxyapatite, (**f**) Joint Committee on Powder Diffraction Standards (JCPDS) no. 090432, and (**g**) gelatin–hyaluronate/calcium sulfate/hydroxyapatite (Gel-HA/CS/HAP) composite.

The XRD pattern of the Gel-HA/CS/HAP composite is shown in Figure 1. Comparing the patterns, the Gel/HA/CS/HAP composite (Figure 1g) had a composition similar to the human femur bone (Figure 1a). There was a broad peak at 2θ = ~20° among the patterns of sodium hyaluronate (Figure 1b), gelatin (Figure 1c), and the Gel-HA/CS/HAP composite (Figure 1g). This characteristic peak was assigned to the crystalline structure in hyaluronic acid and the triple-helical crystalline structure in gelatin. Comparing the patterns of calcium sulfate (Figure 1d) and the Gel-HA/CS/HAP composite (Figure 1g), it was confirmed that the composite did contain calcium sulfate, which did not change in structure after the crosslinking process. Furthermore, comparing the pattern of the hydroxyapatite prepared in this study (Figure 1e) and the standard pattern provided by the Joint Committee on Powder Diffraction Standards (JCPDS) (Figure 1f), the prepared hydroxyapatite was consistent with the standard, and the crosslinking process also did not affect the structure of the hydroxyapatite.

### 3.2. Fourier-Transform Infrared Spectroscopy (FTIR) Analysis

**Figure 2:**
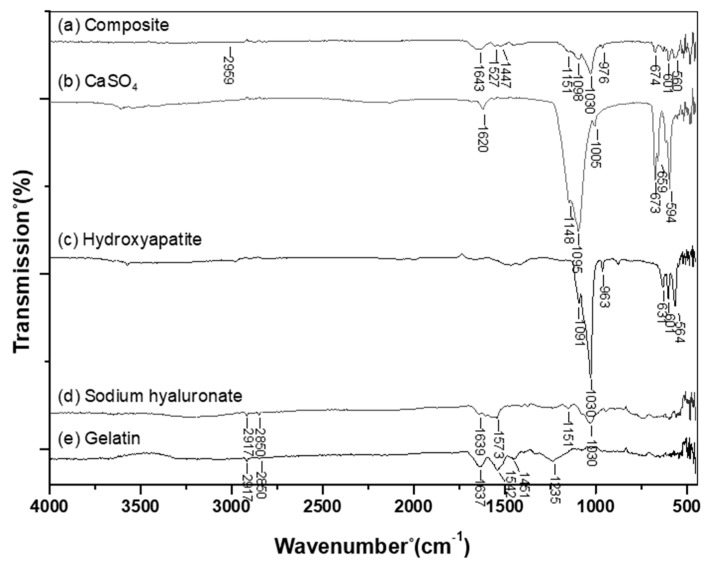
Fourier-transform infrared spectroscopy (FTIR) spectrum: (**a**) Gel-HA/CS/HAP composite, (**b**) calcium sulfate, (**c**) hydroxyapatite, (**d**) sodium hyaluronate, and (**e**) gelatin. The absorbance band at 2959 cm^−1^ was the C–H bonding stretching of 1,4-butanediol diglycidyl ether (BDDE), indicating that the gelatin and hyaluronic acid in the composite of this study were successfully crosslinked.

The results of the FTIR analysis are shown in Figure 2. On the FTIR spectrum of the Gel-HA/CS/HAP composite (Figure 2a) and calcium sulfate (Figure 2b), the absorbance bands of S–O bonding were detected at 1151, 1148, 1098, 1095, 674, and 673 cm^−1^. For the spectrum of hydroxyapatite (Figure 2c), 1091, 1030,963, 631, 601, and 564 cm^−1^ were the absorbance bands of P–O bonding. Among the FTIR spectra of the composite, sodium hyaluronate (Figure 2d), and gelatin (Figure 2e), the absorbance band of amide I C = O bonding could be seen at 1637–1639 cm^−1^, while the absorbance band of amide II N–H and C–N bonding were found at 1520, 1542, and 1537 cm^−1^. For the composite and sodium hyaluronate, 1151 and 1159 cm^−1^ were the absorbance bands of ether C–O–C bonding. The absorbance band of alcohol group C–OH bonding could be seen on the FTIR spectrum of sodium hyaluronate at 1030 cm^−1^. As for the absorbance band of amide III C–N and N–H bonding, it was shown in the spectrum of the composite and gelatin at 1235 cm^−1^.

As previously mentioned, the FTIR spectrum was used to determine the success of crosslinking. Many ether bonds were formed when HA and Gel were crosslinked by BDDE. However, in the HA backbone, there were many ether bonds, so it was not possible to use the absorbance of an ether bond to determine whether the crosslinking process was successful. From the FTIR results of the composite, there was an absorbance band at 2959 cm^−1^. This absorbance band was the C–H bonding stretching of BDDE, which indicated that the Gel-HA/CS/HAP composite was successfully crosslinked.

### 3.3. Swelling Ratio

**Figure 3:**
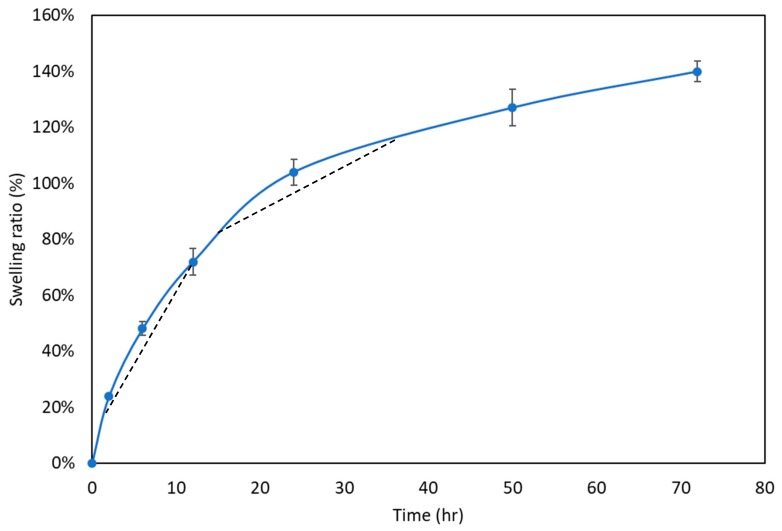
Swelling ratio test (*n* = 6). The Gel-HA/CS/HAP composite performed two-stage swelling. The first stage was the result of hyaluronic acid, and the second stage was caused by gelatin.

As is well known, the swelling of a composite is caused by the absorption of water. According to the results shown in Figure 3, the Gel-HA/CS/HAP composite underwent two-stage swelling. The composite used in this study was copolymerized by Gel and HA, which naturally have different water absorption abilities. Furthermore, Gel conducted less water absorption after being crosslinked. Therefore, the first stage of swelling was attributed to HA, for which water absorption was faster. The second stage of swelling was attributed to Gel since HA already attained its maximum water absorption.

### 3.4. Degradation Rate

**Figure 4:**
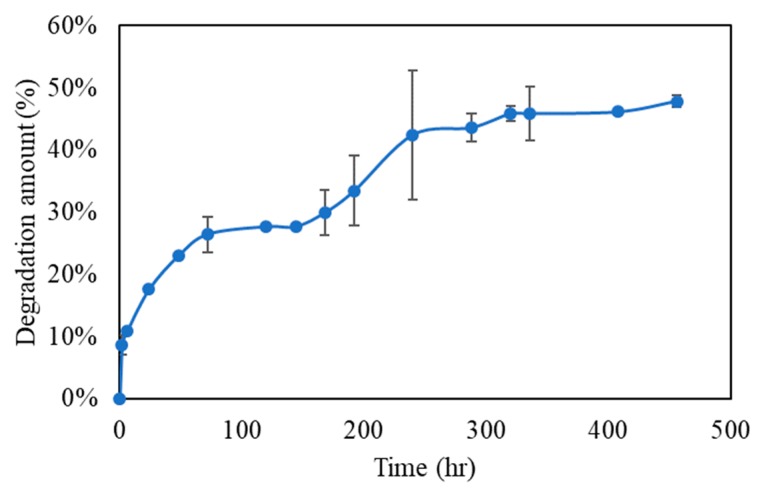
In vitro degradation test (*n* = 6). The Gel-HA/CS/HAP composite underwent two-stage degradation. At the first stage, due to the fact that gelatin did not swell dramatically, little degradation occurred. At the second stage, gelatin absorbed water and swelled considerably, causing a relatively large amount of degradation.

Degradation occurred after the composite absorbed water and swelled. In our study, the Gel-HA/CS/HAP composite exhibited two-stage degradation (Figure 4). From the results of the swelling ratio analysis, Gel initially contributed less to swelling. Therefore, Gel also expressed less degradation at the first stage of composite degradation. After Gel started to swell dramatically, it considerably degraded during the second stage of degradation.

### 3.5. SDF-1 Release Profile

**Figure 5:**
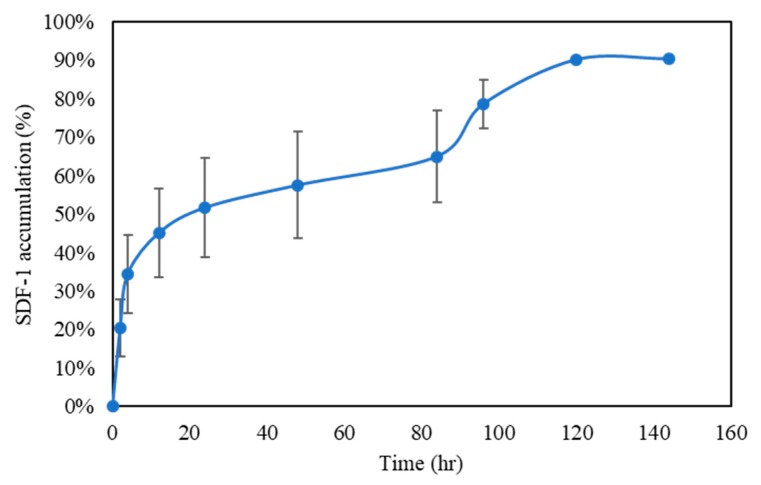
Stromal-cell-derived factor-1 (SDF-1) release profile (*n* = 6). The Gel-HA/CS/HAP underwent a two-stage release. The first stage of release was caused by the SDF-1 molecules at the surface, and the second stage of release was caused by the degradation of the composite.

The release profile of SDF-1 was also divided into two stages (Figure 5). Since SDF-1 was not crosslinked with Gel or HA, the first-stage release was probably caused by SDF-1, which adsorbed at the surface of the Gel-HA/CS/HAP composite. After that, the second-stage release lasted for nearly five days. It was relatively slow and stable, which may have been the result of the degradation of the Gel-HA/CS/HAP composite—mostly the degradation of Gel, followed by the release of SDF-1. According to previous studies, collagen has a binding domain for SDF-1 [32,33,34]. As is known, HA is a polysaccharide and Gel is a denatured collagen. Therefore, Gel might have a higher affinity to SDF-1 than HA. The course of SDF-1 release lasted for five days in total, and the effective release was about 90%.

### 3.6. Cell Viability

**Figure 6:**
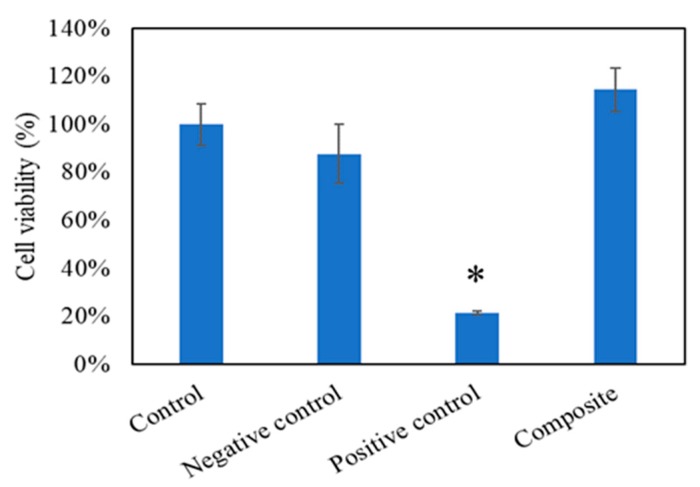
WST-1 test (*n* = 6). Control group was 100% cell viability, negative control was aluminum oxide, and positive control was zinc diethyldithiocarbamate. The results showed that the Gel-HA/CS/HAP composite had no cell toxicity. * *p* < 0.001.

Cell viability and proliferation were determined by a WST-1 assay. cbMSC-hTERT cells were treated with the medium extracts of the negative control (aluminum oxide, Al_2_O_3_), positive control (zinc diethyldithiocarbamate, ZDEC), and the Gel-HA/CS/HAP composite, respectively, at the concentration of 0.2 g/mL. The control group (nontreated) was defined as 100% cell viability. As expected, the negative control and Gel-HA/CS/HAP composite did not affect the growth of cbMSC-hTERT cells (Figure 6). However, the positive control did inhibit the growth of cbMSC-hTERT cells.

### 3.7. MSC Recruitment Test

**Figure 7:**
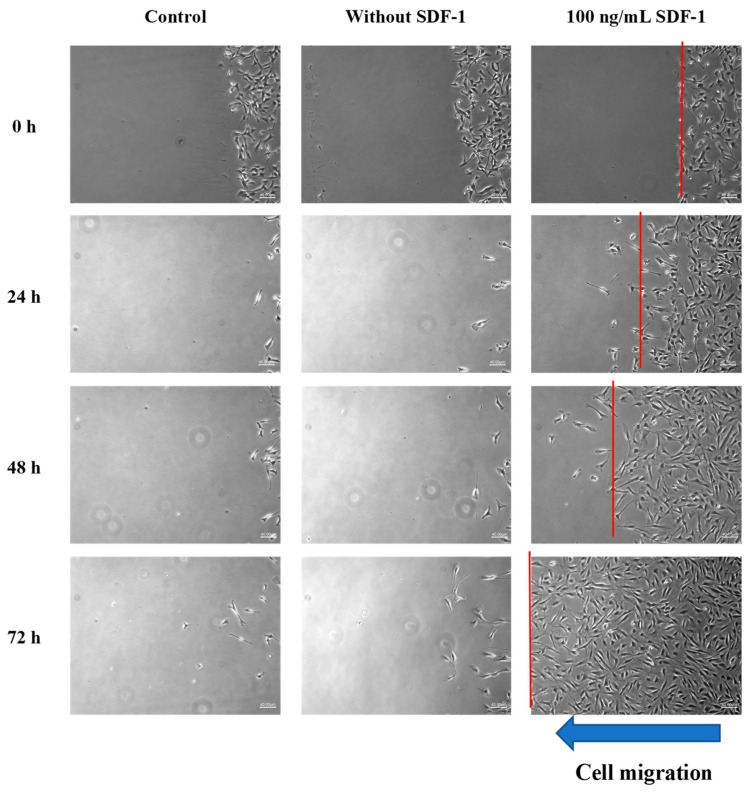
Mesenchymal stem cell (MSC) recruitment test (40×). First, second, and third columns show MSC migration of the control group, without SDF-1 group, and the 100 ng/mL SDF-1 group, respectively. The red line indicates the cell front. The results show that the MSCs of the control group and without SDF-1 group gathered together and formed colonies. MSCs of the 100 ng/mL group migrated toward the concentration gradient of SDF-1.

**Figure 8:**
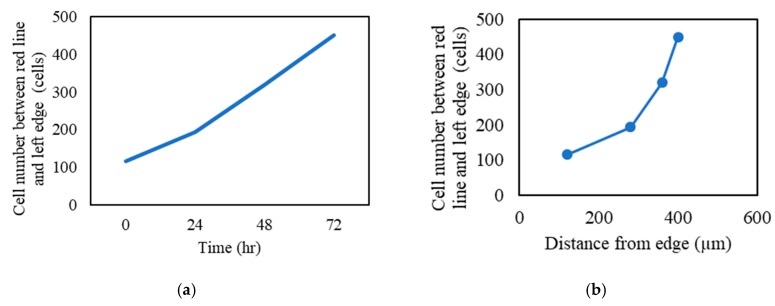
MSC recruitment test analysis: (**a**) cell number between the red line and left edge versus time and (**b**) cell number between the red line and left edge versus distance from the left edge.

In order to confirm the SDF-1 chemotaxis ability of the composite, an MSC recruitment test was conducted. cbMSC-hTERT cells were placed at the right and the material was placed at the left in the beginning (0 h) in all groups (Figure 7). After 24 h, the cbMSC-hTERT cells of the control group and the group without SDF-1 started to form colonies due to its fibroblast-like nature. However, the cbMSC-hTERT cells did show some cell migration from right to left in the 100 ng/mL SDF-1 group, which was related to the SDF-1 concentration gradient released from the Gel-HA/CS/HAP composite on the left side. After 48 and 72 h, the phenomena of each group became more apparent. According to the cell-number–time analysis, cbMSC-hTERT cells moved toward the Gel-HA/CS/HAP/SDF-1 composite as the time increased (Figure 8a). From the cell-number–distance analysis, cbMSC-hTERT cells migrated toward the Gel-HA/CS/HAP/SDF-1 composite as the distance increased from the left side (Figure 8b). All these results indicated that the Gel-HA/CS/HAP/SDF-1 composite was capable of attracting MSCs. Moreover, SDF-1 did not change the fibroblast-like type of MSCs or have any impact on MSCs.

### 3.8. In Vivo Study

In the in vivo study, no infection or severe side effects were observed at implant sites and no deaths were recorded during the recovery period. Soft tissue wounds recovered without showing any acute inflammation in experimental, control, and comparison groups.

#### 3.8.1. Blood Analysis

Before sacrifice, cardiac puncture was done for biochemical and whole blood analyses, and the results were compared with standard range (Charles River Laboratories, 1982). The biochemistry tests after one and two months are shown in Table 1 and Table 2. As we can see, the Ca values of all groups were in a normal range. LDH values of the 100 ng/mL SDF-1 group after two months were slightly higher than the normal range but were still acceptable. The results of the whole blood tests are shown in Table 3 and Table 4. The WBC values of all groups were elevated slightly after one month but returned to normal after two months, which may have been related to the inflammatory response caused by the created bone defects. Based on the results of the biochemical and whole blood analyses, the Gel-HA/CS/HAP composites with or without SDF-1 prepared in this study appeared to have no obvious systemic toxicity.

#### 3.8.2. Two-Photon Excitation Microscopy

**Figure 9:**
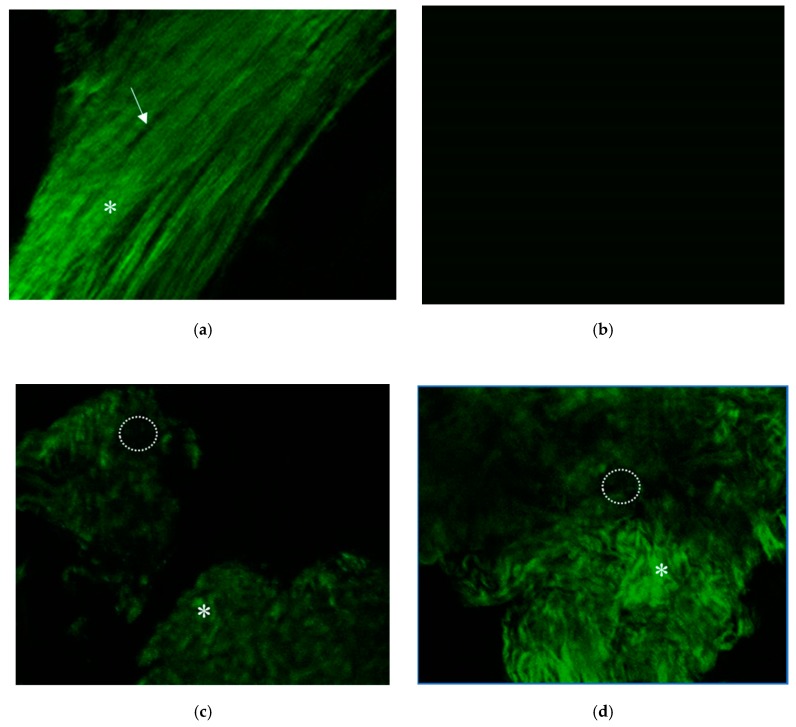
Two-photon excitation microscopy. (**a**) Second harmonic generation (SHG) signals of normal rat femur bone tissue. Green woven fibers (white asterisk) indicate well-aligned type I collagen fibers of mature bone matrix. Dark spindle-shaped areas (white arrow) in this image might present the location of osteocytes. (**b**) SHG signals of Gel-HA/CS/HAP composite before implantation. (**c**) SHG signals of the drill hole site filled with the composite without SDF-1. Some woven type I collagen fibers (asterisk) can be found, which indicate the evidence of new bone formation around the bone defect site. The dark areas (dotted circle) in this picture might be those osteocytes that just migrated in or the residual CS/HAP particles. (**d**) SHG signals of bone defect site implanted with the composite with 100 ng/mL SDF-1. Stronger signals of collagen fibers can be seen.

Second harmonic generation (SHG) is an optical phenomenon in which two incident photons are converted into a single photon by interacting with the crystalline optical structure of some materials. In biological tissues, type I collagen is known to have strong SHG signals. We used a two-photon microscope with 840 nm excitation and a 420/20 nm bandpass filter to detect SHG signals from type I collagen in the specimens. Fresh femur specimens and composites were immersed in normal saline for inspection using a 20× water immersion lens. Figure 9a shows the normal rat femur bone structure under the two-photon microscope. The green woven fibers present well-aligned type I collagen fibers of the mature bone matrix, and the dark spindle-shaped areas might indicate the location of osteocytes. Figure 9b shows the SHG signals of the composite before implantation. The image was totally dark because there was no collagen inside. Figure 9c shows the SHG signals of the drill hole site which was implanted with the Gel-HA/HAP composite without SDF-1 for one month. Compared with the normal mature bone structure, the type I collagen fibers were looser and not so well aligned, which indicated the evidence of new bone formation inside the created bone defect. The dark areas in this picture might be the newly migrated osteocytes or residual HAP particles. Figure 9d shows the SHG signals of the drill hole site which was implanted with the Gel-HA/HAP composite with 100 ng/mL SDF-1. The signals of collagen fibers in composite with the SDF-1 group seem to be stronger then signals in composite without the SDF-1 group. Many various sized dark areas can also be seen between collagen fibers.

In this study, SHG imaging was used to confirm the bone ingrowth of the defect site filled with composites in an animal model. After a literature review, we found only a few articles about using two-photon excitation microscopy or SHG imaging on bone structure [35,36,37]. Due to the limited penetration depths of the laser beam, the complete bone structure cannot easily be visualized. Ishii et al. demonstrated dynamic live imaging of animal bone tissue under two-photon microscopy after injection of various fluorescent agents [35]. According to their study, both static histological information and the dynamic behaviors of live bone cells can be recorded under two-photon microscopy. In our study, however, no fluorescent agent was needed. Thanks to the SHG signals from type I collagen fibers, we could still successfully observe the normal bone structure of fresh rat femurs and the new bone growth on implanted composites. Using this technique, live SHG imaging of bone growth under two-photon microscopy may be possible in the future.

#### 3.8.3. Micro-CT

**Figure 10:**
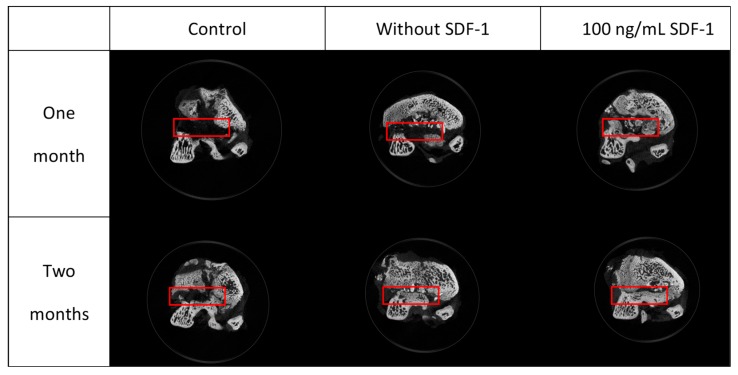
Micro-CT sections of specimens. Red rectangles indicate the site of the bone defect created by the drill. More bone ingrowth can be found in composite groups, especially the group with SDF-1.

**Figure 11:**
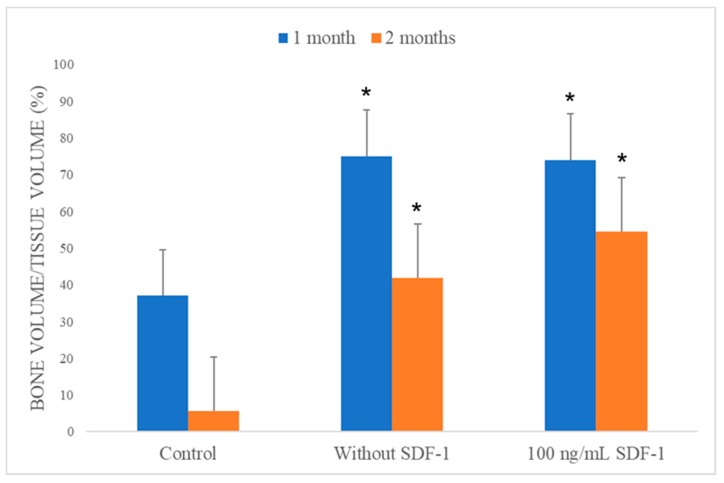
Bone volume/tissue volume (BV/TV) analysis of the scanned samples (*n* = 3). An obvious increase of new bone formation was noted in the composite without and composite with 100 ng/mL SDF-1 groups compared with the control group. * *p* < 0.05, compared with control group.

The area of the bone defect site of each specimen was labeled under micro-CT sections (Figure 10). The percentages of new bone formation in each defect area were analyzed by CTan software and presented as BV/TV ratios (Figure 11). As we can see, although the bone had the ability of self-healing, regeneration in the control group was limited. Under the micro-CT section, the defect sites in the control group were still quite obvious after both one and two months. The BV/TV ratios of the control group after one and two months were 37.07% and 5.65%, respectively. For the micro-CT sections in the without SDF-1 group, some residual materials of the composites could still be seen, and there was more new bone formation in the defect area compared with the control group. The BV/TV ratios of the without SDF-1 group after one and two months were 75.06% and 41.89%, respectively. As for the 100 ng/mL SDF-1 group, its micro-CT sections revealed more bone formation compared with the other two groups. The BV/TV ratios of the percentages of the 100 ng/mL SDF-1 group after one and two months were 74.05% and 54.52%, respectively.

As we can see, the composites in both the without SDF-1 and 100 ng/mL SDF-1 groups were absorbed gradually. The results of the swelling and degradation tests suggest that after implantation, swelling resulted in material degradation, leading to absorption by cells. In addition, as it was shown in our micro-CT results, both composite groups without SDF-1 and with 100 ng/mL SDF-1 significantly had better bone healing compared with the control group. Compared with the composite without SDF-1 group after two months, the 100 ng/mL SDF-1 group showed a trend of better bone healing, but it did not achieve significance statistically. The probable reasons why it failed to show a significant difference between two kinds of composite include the limited number of experimental animals and suboptimal dose of SDF-1. In summary, the results of micro-CT analysis showed that Gel-HA/CS/HAP composites can enhance bone regeneration in an animal bone defect model. These results also implied that the MSC migration promoted by SDF-1 may be beneficial for bone healing, which still requires more studies to prove.

#### 3.8.4. Histological Analysis

**Figure 12:**
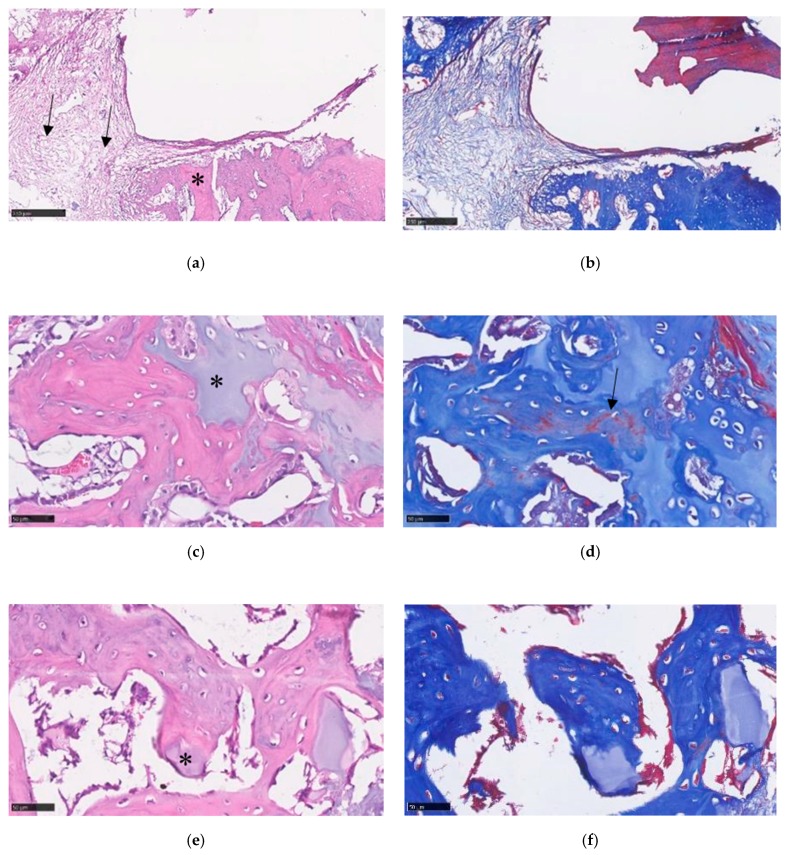
(**a**) Histology section under 10× magnification view showed a large bone defect and normal bone structure (asterisk) in the control group under hematoxylin and eosin (H&E) staining. Some fibrotic tissue (black arrows) was also found in the bone defect. (**b**) No new bone formation was noted under Masson’s trichrome (MT) stain in the control group. (**c**) Under 40× magnification view of the composite without SDF-1 group, residual material (asterisk) can be seen. (**d**) New bone formation (black arrow) can be found under MT stain in the group without SDF-1. (**e**) In the 100 ng/mL SDF-1 group, smaller residual material (asterisk) was shown to be well integrated into the normal bone structure under H&E stain. (**f**) Histology section of SDF-1 group under MT stain.

For histological analysis, the H&E and MT stain of the control group showed obvious bone defects even after two months (Figure 12a,b). Some fibrotic tissue ingrowth was also noted, and no new bone formation was found around the defect sites under MT stain. Histology sections of groups adding Gel-HA/CS/HAP composites with or without SDF-1 showed new bone formation under MT stain (Figure 12d,f). Residual materials were noted in both composite groups. Under H&E stain of both with and without SDF-1 groups, the residual materials were well integrated with normal bone tissue under MT stain (Figure 12c,e). Furthermore, the size of the residual material seemed to be smaller in the group with 100 ng/mL SDF-1 compared with the group without SDF-1.

In a study by Faruq et al. [38], biphasic calcium phosphate (CP) granules were combined with HA-Gel in a rabbit model, and promising bone regeneration was demonstrated by their CP/HA-Gel compounds in rabbit femur bone defects. An HAP/CS/HA composite encapsulated with collagenase (Col) was introduced by Subramaniam et al. for rat alveolar bone defects [39]. In their study, this HAP/CS/HA-Col composite improved new bone formation in a rat alveolar model. The use of a Gel-modified CS-HAP bone void filler with the addition of BMPs and bisphosphonate in a rat tibia bone defect model was reported by Teotia et al. [40]. Gelatin cement resulted in better cell proliferation in their study compared with cement without gelatin. In their rat alveolar defect model, maximum bone formation was found in animals implanted with cement incorporated with zoledronic acid. In our study, we integrated particles of calcium sulfate and hydroxyapatite directly into gelatin and hyaluronic acid as a composite. A rat femur bone defect model created by a trephine drill was used for our animal study. Comparable results were also achieved in our study, despite some differences in the study designs. Furthermore, there was no obvious systemic toxicity noted from our blood test results which could have been caused by the Gel-HA/CS/HAP composites with or without SDF-1. All the results showed that the use of Gel-HA/CS/HAP composites in bone defects seems to be safe and feasible, at least in a rat animal model.

The exact role of SDF-1 in bone formation remains unclear. As previously mentioned, in several studies, SDF-1 was found to have the ability to recruit MSCs [17,18,19]. Also, previous reports have shown the potential of MSCs in bone regeneration enhancement [17,20,21]. Even though the mechanism of MSCs during bone regeneration is not yet fully understood, we thought that the recruitment of MSCs may provide a certain degree of osteoinductive effect in the bone healing process—something which may require further investigation to confirm [41]. Shen et al. used a silk fibroin–nanohydroxyapatite scaffold with sustained release of SDF-1 and BMP-2 on a rat cranial bone defect model [42]. They successfully proved that combining SDF-1 and BMP-2 results in a synergistic effect on bone regeneration. In our study, cbMSC-hTERT cells also obviously migrated toward SDF-1-releasing material compared with the control groups. Furthermore, adding SDF-1 into the Gel-HA/CS/HAP composite showed comparable results to the pure composite group and even revealed a trend toward better bone regeneration.

The size, hardness, and applicability of bone void fillers are quite important when dealing with bone defects in clinical practice [3,4]. It is important that the materials be easy to manipulate and handle during the implantation of bone fillers into bone defect sites. When the material is too large, it may require more time to fabricate or trim it to the correct size before implantation. Also, it may be difficult to implant into the bone defect if the material is too hard. However, if the material is too small or too soft, the material may be easily spread out around the operation field during implantation. In that situation, heterotrophic bone formation or soft tissue irritation may develop. In this study, the Gel-HA/CS/HAP composite had adequate but not excessive hardness and could be easily cut into a suitable size without difficulty. The composite could be handled and implanted into the bone defect site without it spreading out. Nevertheless, the composite could be retained inside the bone defect site without dissolution or dislocation, even after saline or blood flushes, which is quite important during real operations on humans. The good applicability and sustainability of this composite appears to make it suitable for clinical use.

There are some limitations to our study. Statistical analysis was difficult because of the small number of animal models. Furthermore, due to the fast bone generation speed of rats, shorter observation intervals may be needed for more detailed comparison. In this study, human SDF-1 was used on the rat model for the in vivo test. According to a previous study, human and murine SDF-1 are highly similar and can react across species [43]. However, the MSC recruitment ability of human SDF-1 may still possibly be compromised in a rat model.

## 4. Conclusion

In summary, the good biocompatibility and biodegradability of Gel-HA/CS/HAP composites with or without SDF-1 were both confirmed by in vitro tests in our study. Enhanced bone regeneration after implantation of Gel-HA/CS/HAP composites was also observed in micro-CT and histology analyses in our in vivo study. The composites with SDF-1 showed a trend of better bone healing compared with the composite without SDF-1, but the difference was not statistically significant. In conclusion, this Gel-HA/CS/HAP composite with SDF-1 may be a safe and feasible material to use as a bone void filler in bone defects. Further studies are still needed to reveal the definite pathway by which SDF-1 enhances bone growth and the best dose of SDF-1 which should be added to the composite.

## Figures and Tables

**Table 1:** Biochemistry test (one month).

	Control	Without SDF-1	With SDF-1	Reference *
ALKP (U/L)	310.67	276	253	39–216
Ca (mg/dL)	11.63	12.1	12.27	8–15
LDH (U/L)	656.33	459.33	430	300–700

* Charles River Laboratories, 1982. ALKP: alkaline phosphatase; LDH: lactate dehydrogenase.

**Table 2:** Biochemistry test (two months).

	Control	Without SDF-1	With SDF-1	Reference *
ALKP (U/L)	260.33	214.67	214.67	39–216
Ca (mg/dL)	10.97	10.77	10.83	8–15
LDH (U/L)	586	368.67	771	300–700

* Charles River Laboratories, 1982. ALKP: alkaline phosphatase; LDH: lactate dehydrogenase.

**Table 3:** Whole blood test (one month).

	Control	Without SDF-1	With SDF-1	Reference *
RBC (M/µL)	8.32	8.45	8.3	7.37–9.25
HGB (g/dL)	14.73	15.3	15.1	14.4–17.6
HCT (%)	44.4	46.37	45.63	36–46
MCV (fL)	53.37	54.83	54.97	47–52
MCH (pg)	17.7	18.1	18.2	17–21
MCHC (g/dL)	33.2	33	33.1	35–43
WBC (K/µL)	11.52	13.99	12.33	6.19–12.55
NEUT (%)	16.67	14.13	19.23	1–29
LYMPH (%)	75.27	80.9	73.57	70–99
MONO (%)	5.23	2.97	5.13	0–6
EO (%)	2.63	1.9	2	0–3
BASO (%)	0.2	0.1	0.07	0–2

* Charles River Laboratories, 1982. RBC: red blood cell; HGB: hemoglobulin; HCT: hematocrit; MCV: mean corpuscular volume: MCH: mean corpuscular hemoglobin; MCHC: mean corpuscular hemoglobin concentration; WBC: white blood cell; NEUT: neutrophil; LYMPTH: lymphocyte; MONO: monocyte; EO: eosinophil; BASO: basophil.

**Table 4:** Whole blood test (two months).

	Control	Without SDF-1	With SDF-1	Reference *
RBC (M/µL)	9.16	9.18	8.87	7.37–9.25
HGB (g/dL)	15.47	15.4	15.00	14.4–17.6
HCT (%)	45	45.47	43.75	36–46
MCV (fL)	49.14	49.58	49.35	47–52
MCH (pg)	16.90	16.79	16.92	17–21
MCHC (g/dL)	34.37	33.89	34.29	35–43
WBC (K/µL)	10.29	10.33	10.05	6.19–12.55
NEUT (%)	16.67	14.13	19.23	1–29
LYMPH (%)	75.27	80.9	73.57	70–99
MONO (%)	5.23	2.97	5.13	0–6
EO (%)	2.63	1.9	2	0–3
BASO (%)	0.2	0.1	0.07	0–2

* Charles River Laboratories, 1982. RBC: red blood cell; HGB: hemoglobulin; HCT: hematocrit; MCV: mean corpuscular volume: MCH: mean corpuscular hemoglobin; MCHC: mean corpuscular hemoglobin concentration; WBC: white blood cell; NEUT: neutrophil; LYMPTH: lymphocyte; MONO: monocyte; EO: eosinophil; BASO: basophil.
